# Applications of Mixed Reality Technology in Orthopedics Surgery: A Pilot Study

**DOI:** 10.3389/fbioe.2022.740507

**Published:** 2022-02-22

**Authors:** Lin Lu, Honglin Wang, Pengran Liu, Rong Liu, Jiayao Zhang, Yi Xie, Songxiang Liu, Tongtong Huo, Mao Xie, Xinghuo Wu, Zhewei Ye

**Affiliations:** ^1^ Department of Orthopaedics Surgery, Union Hospital, Tongji Medical College, Huazhong University of Science and Technology, Wuhan, China; ^2^ Intelligent Medical Laboratory, Union Hospital, Tongji Medical College, Huazhong University of Science and Technology, Wuhan, China; ^3^ Department of Orthopaedic Surgery, Puren Hospital of Wuhan, Wuhan University of Science and Technology, Wuhan, China

**Keywords:** mixed reality (MR), augmented reality (AR), orthopaedics, surgical visualization, workload, telesurgery, navigation, cloud platform

## Abstract

**Objective:** The aim of this study is to explore the potential of mixed reality (MR) technology in the visualization of orthopedic surgery.

**Methods:** The visualization system with MR technology is widely used in orthopedic surgery. The system is composed of a 3D imaging workstation, a cloud platform, and an MR space station. An intelligent segmentation algorithm is adopted on the 3D imaging workstation to create a 3D anatomical model with zooming and rotation effects. This model is then exploited for efficient 3D reconstruction of data for computerized tomography (CT) and magnetic resonance imaging (MRI). Additionally, the model can be uploaded to the cloud platform for physical parameter tuning, model positioning, rendering and high-dimensional display. Using Microsoft’s HoloLens glasses in combination with the MR system, we project and view 3D holograms in real time under different clinical scenarios. After each procedure, nine surgeons completed a Likert-scale questionnaire on communication and understanding, spatial awareness and effectiveness of MR technology use. In addition to that, the National Aeronautics and Space Administration Task Load Index (NASA-TLX) is also used to evaluate the workload of MR hologram support.

**Results:** 1) MR holograms can clearly show the 3D structures of bone fractures, which improves the understanding of different fracture types and the design of treatment plans; 2) Holograms with three-dimensional lifelike dynamic features provide an intuitive communication tool among doctors and also between doctors and patients; 3) During surgeries, a full lesion hologram can be obtained and blended in real time with a patient’s virtual 3D digital model in order to give surgeons superior visual guidance through novel high-dimensional “perspectives” of the surgical area; 4) Hologram-based magnetic navigation improves the accuracy and safety of the screw placement in orthopaedics surgeries; 5) The combination of mixed reality cloud platform and telemedicine system based on 5G provides a new technology platform for telesurgery collaboration. Results of qualitative study encourage the usage of MR technology for orthopaedics surgery. Analysis of the Likert-scale questionnaire shows that MR adds significant value to understanding and communication, spatial awareness, learning and effectiveness. Based on the NASA TLX-scale questionnaire results, mixed reality scored significantly lower under the “mental,” “temporal,” “performance,” and “frustration” categories compared to usual 2D.

**Conclusion:** The integration of MR technology in orthopaedic surgery reduces the dependence on surgeons’ experience and provides personalized 3D visualization models for accurate diagnosis and treatment of orthopaedic abnormalities. This integration is clearly one of the prominent future development directions in medical surgery.

## Introduction

Understanding the patient-specific three-dimensional (3D) anatomy is essential for both preoperative planning and intraoperative decision making. Although conventional 3D image data such as mixed reality (MR) and computerized tomography (CT) images offer excellent discriminatory properties, these 3D volumetric images are still displayed in 2D on a flat screen, limiting the real depth perception and the possibilities for interaction in 3D space (Kumar et al., 2020; Lungu et al., 2021). Two-dimensional visualization of 3D datasets still leaves a lot of the understanding of complex anatomical structures to the spatial imagination of individuals, which often makes it difficult for surgeons to clarify detailed information of lesions and their nearby anatomical structures (Brun et al., 2019). By using these flattened 3D datasets for preoperative assessment and planning, surgeons are forced to create 3D models in their minds with obvious potential flaws, which may not only affect the development of the surgical strategy, but may also result in a failure to deliver effective surgical information to the patient during the decision-making and consent-signing processes. In addition, seamless integration of the 3D images into the actual surgical procedure is difficult (Sauer et al., 2017). At this stage, there is still a spatial and temporal separation between the image and the surgical act. Intraoperatively, the surgeon needs to constantly switch her attention between the surgical area and the image monitor, mentally reconstructing the image and translating it to the same orientation and position as the patient. This lack of synchronization between the images and the surgical field results in a lack of hand-eye coordination, which may in turn affect the surgeon’s performance (Tepper et al., 2017). Mixed reality (MR) technology has recently provided simple and easy-to-use solutions to simulate 3D images as well as reduce the bias between the workspace and visualization.

In fact, artificial reality technologies, which include virtual reality (VR), augmented reality (AR), and mixed reality (MR), have sought to address 3D visualization needs in medicine since the early 1990s (Brun et al., 2019; Carbone et al., 2020; Lungu et al., 2021). Among artificial reality technologies, VR has been defined as a technology that immerses the user in a completely artificial, computer-generated environment (Brigham, 2017). AR is a technology which superimposes digital with real objects and permits interaction with both types of objects (Carbone et al., 2020; Condino et al., 2021b). Although both AR and VR show promise, there are severe limitations that have prevented their broad application in surgical field. These include the inability to perceive the depth and perspective of the virtual elements in AR, and exclusion of the real-world environment in VR. The concept of mixed reality, which is also known as “reality-virtual continuum” ranging from the completely real to a completely virtual environment, was proposed in 1994 (Milgram and Kishino, 1994; Goo et al., 2020). As a new-generation reality technology, MR technology is a combination of virtual reality (VR) and augmented reality (AR) in 3D applications (Li et al., 2018; Park et al., 2020). A core feature of MR technology is to introduce and integrate 3D hologram into the real world seen by users, and establishes interactive feedback loop between the virtual world and the real world to enhance the sense of reality and space of user experience. (Lee et al., 2016; Martin et al., 2020) (Duff et al., 2013; Schuster-Amft et al., 2014; Elliott et al., 2015; Colomer et al., 2016). Compared to AR and VR, MR not only anchors virtual objects into the physical world, enabling the user to interact with both virtual and real objects, but also allows the user to experience depth and perspective, which significantly improves 3D visualization of surgical anatomy. (Wu et al., 2018a; Brun et al., 2019; Chytas and Nikolaou, 2021). MR holograms have several advantages as an assistive tool in surgical procedures (Halic et al., 2010; Sauer et al., 2017). Namely, 1) real-time sharing of 3D holograms, 2) depth fitting between real and virtual worlds, and 3) real-time interaction. While the usefulness of MR technology has been reported in various medical interventions, including neurosurgical procedures (Comeau et al., 2000), cardiothoracic surgery (Brun et al., 2019), laparoscopic and endoscopic surgery (Al Janabi et al., 2020; Li et al., 2020), there is a dearth of systematic reports and studies on MR technology in orthopaedics surgery, especially studies that provide a detailed clinical-use workflow of MR hologram in different scenarios during orthopaedics surgery from the data capture to holographic visualization to help readers replicate the work. Furthermore, little attention has been paid to get feedback from participants in MR experiments to better understand such technology, despite the increasing interest to bring MR into surgery.

Therefore, in this work, we report in detail preliminary experience in integrating MR technology into orthopaedics surgical workflow under different case scenarios, including preoperative planning, preoperative communication, intraoperative guidance, surgical navigation, and telesurgery consultation. Qualitative tests were also carried out to evaluate workload and usability of MR technology for orthopaedics surgery and to explore advantages and limits of this novel technology.

## Methods

### The Construction of the Orthopaedic Mixed-Reality Surgery System

The proposed mixed-reality system (Visual 3D, Beijing,China) is composed of both hardware and software components. The hardware components include a data-processing computer with a 1.6 GHz processor, a USB electronic encryption dog, HoloLens glasses (Microsoft Corporation, Redmond, WA), a router, a background server and a 3D-imaging workstation. The workstation is mainly composed of four modules: a local database module, a 3D display module, a floating-point function module, and a scene-editing module. The software part includes components for medical image processing, the HoloLens client, and the Pad control terminal. The system has a cloud platform, through which all image data and image processing results can be stored, transmitted and shared. The data-processing computer is used for image enhancement, segmentation, 3D reconstruction as well as virtual surgery simulation of DICOM data acquired by various imaging devices. Here, the abbreviation DICOM stands for Digital Imaging and Communications in Medicine, which is a standard format to view, store, retrieve and share medical data. The MR space-station consists of a mixed-reality display device (HoloLens glasses) and a tablet-computer control device for holographic presentation of 3D anatomical models. The study was approved by the ethics committees of Union Hospital, Tongji Medical College, Huazhong University of Science and Technology.

### Key System Modules

#### Data Collection and 3D Reconstruction

Patient-specific raw data samples, acquired using CT and MRI modalities, were obtained from the picture archive and communication system (PACS) of Wuhan Union Hospital. The data samples were imported into the 3D imaging workstation, StarCloud workstation (a 3D reconstruction software from Visual3D, Beijing,China). This imaging framework employs accurate and fast advanced algorithms (including deep learning algorithms) with flexible manual operations. This enables quick extraction and reconstruction of the target DICOM voxels into a 3D mesh model (STL format) , which can be directly published as a HoloLens holographic scene or a digital asset exchange (DAE) file adapted to the zSpace environment. Finally, the proposed framework embodies fast, accurate and safe 3D reconstruction and analysis of patient image data. The framework also combines private cloud and space-station units to form a closed-loop data-flow scheme involving image data acquisition and analysis, cloud storage and graphics-processing-unit (GPU) rendering, mixed-reality holographic browsing, recording and sharing. This stable imaging scheme enables effective medical treatment through well-designed self-tuned artificial intelligence and machine learning approaches, optimized equipment functions, and enhanced user experiences.

#### Cloud-Based 3D Model Storage and Rendering

A private cloud network ensures smooth and safe use of devices in a local area network (LAN). We established a LAN within our hospital environment using a router to connect workstations, space stations and the cloud infrastructure. The established LAN ensures secure image-data flow in the hospital environment. After the 3D model storage and rendering are completed in the cloud, models are transmitted to the space station through a high-speed LAN. While the space-station devices are limited in memory and computing power, the cloud GPU greatly reduces the computational intensity of these devices, exploits cloud capabilities to improve rendering of voxel-based models, and hence enables real-time smooth viewing of more realistic and clear 3D models.

#### Mixed-Reality Holographic Imaging

All of reconstructed files are imported into the editing module inside the StarCloud workstation for colorization and transparentization, and were saved as .fbx files recognized by MR display device (Microsoft HoloLens) for holographic visualization. Manual segmentation was also performed to highlight relevant structures. Then, holographic 3D models can be spatially viewed with HoloLens. The operator can scale, rotate and move the 3D hologram by gesture or voice control. Moreover, the 3D models can be edited over the Internet, and can also be shared with others synchronously and remotely. At the same time, with a third-angle recording operation, many people can share the holographic view of an interacting doctor.

### Experimental Protocols

To evaluate the usability of the proposed MR visualization system in orthopaedics surgery, a pilot user study has been conducted. The MR system was applied in different clinical scenarios of orthopaedics surgery, including preoperative communication, intraoperative guidance, surgical navigation and remote consultation. After each operation, the surgeons completed a Likert-scale questionnaire on communication and understanding, spatial awareness and effectiveness of MR technology use. NASA Task Load Index (NASA-TLX) were employed to evaluate the workload of intraoperative system support.

### Preoperative Communication/Planning

Preoperatively, raw cross-sectional DICOM images (such as MRI and CT) were segmented and processed to a 3D mesh using StarCloud workstation. After removing the noise and unimportant tissue structures in medical images, the segmented data were converted to a virtual 3D model, then exported into HoloLens headset to produce holograms. In preoperative communication/planning, surgeons, nurses, patients and their families share the 3D hologram of the lesion site and communicate treatment options by wearing HoloLens. These users not only can observe the hologram from equal or different angles, but also interact simultaneously using the manipulation tools.

### Intraoperative Guidance

During the operation, all surgeons put on HoloLens glasses for real-time sharing of the patient’s hologram through the interaction between the MR system and the patient-specific case. Using the HoloLens, projecting a virtual holographic model into the actual superficial anatomy of a patient or just above it to avoid obstructing a surgeon’s view. The 3D virtual model is manually aligned with the patient’s body by registering the major anatomical landmarks. At this point, a surgeon can perform surgery while observing virtual 3D models to guide the design of skin incisions, the angle of screw placement, the range of tumor resection, and so on.

### Surgical Navigation

The MR navigation system is mainly based on TrakStar, which is a high-accuracy electromagnetic tracker designed for short-range motion tracking applications. System components of the navigation system were the following: TrakStar tracking system, a flat-panel monitor, an electro-magnetic transmitter, six degrees-of-freedom (6DOF) sensors and electronics unit. The 6DOF sensor provides a tracking solution that includes the position in three dimensions and the orientation of the three sensor axes relative to the tracker reference frame. As part of the 3D Guidance product suite, the trakSTAR electromagnetic tracking system provides high-precision, real-time unobstructed tracking of miniature sensors embedded in medical tools. To track the position of TrakStar tracked pointing tool, the TrakStar tracking tool (sensor) was mounted near the surgical area and used as a reference coordinate system. The virtual model was imported to HoloLens glasses and utilized in conjunction with navigation for enhanced guidance via MR hologram overlay with the surgical area. By integrating the electromagnetic signals and virtual model, the MR navigation could provide accurate and flexible guidance during orthopaedics surgery.

### Telesurgury Consultation

The MR-based telesurgery system interconnectes an operation site and a guidance site via a public 5G wireless network, which provided bidirectional data transmission connecting both ends of the system.The 5G Customer Premises Equipment (CPE) was used as a terminal receiver for the 5G radio network signals generated by the 5G base station. Surgical data was also transmitted from the 5G CPE to the 5G base station. The data was then transmitted from this station to the destination station *via* the 5G core network. In this study, the bandwidth of the 5G wireless network was 1 Gb/s. For real-time communication and coordination, the two sites were also connected *via* a video conferencing system and a 4 K resolution screen (Hisense). In addition, the 3D holographic models reconstructed by MR system were separately displayed on another 4 K resolution screen. The operation field and the expert real-time guidance scenes are captured by HD cameras. Then, the images are integrated and displayed to realistic on-site collaboration among doctors at different locations. With the support of MR technology and 5G wireless network, external specialists can share 3D holograms with local doctors for remote surgical consultation. During surgery, doctors at different locations can easily communicate in real time, and mark structures within the surgical site with dots, lines, or arrows using a stylus on networked computer screens via augmented reality technology. The MR hologram could be controlled by the experts to send instructions and virtual operations to the surgeons. On this basis, operations can be performed safely, accurately and efficiently. The proposed MR-based telesurgery system makes remote surgical consultation a reality by virtual real-time presence of off-site experts into the surgical theatre. A schematic of 5G-assisted MR telesurgury consultation can be found in Supplementary Figure S1.

### A Questionnaire Survey on Mixed Reality

The method of the National Aeronautics and Space Administration-Task Load Index (NASA-TLX)(Patel et al., 2020; Said et al., 2020) was used to evaluate the clinical workload of MR technology. The load index is based on six items, namely mental demand (“How mentally demanding was the task?”), physical demand (“How physically demanding was the task?”), temporal demand (“How hurried or rushed was the pace of the task?”), effort (“How hard did you have to work to achieve your level of performance?”), performance (“How successful were you in performing the task?”) and the frustration level (“How insecure, discouraged, irritated, stressed, and annoyed were you?”). This index was used to investigate the workload of nine surgeons who had interacted with MR modules in our test environment. These MR modules were compared to traditional 2D display technologies using a paper questionnaire with relevant information. The NASA-TLX score is scaled to be in the range of 0–100 in order to facilitate comparison ^[20]^. The higher the score, the greater the total load. Moreover, a Likert-scale questionnaire (with possible responses typically ranging from 1 for “strongly disagree” to 5 for “strongly agree”) that assessed their opinions on the effect the 3D hologram had on understanding and communication, spatial awareness, and effectiveness as surgical supporting tool.

### Statistical Analysis

Statistical analyses were performed using the GraphPad prism, version 8 (GraphPad Software) and SPSS version 20.0 (Statistical Package for the Social Sciences, IBM). Continuous variables were expressed as mean ± standard deviation (SD), medians and interquartile ranges or simple ranges, as appropriate data was processed using analysis of variance (ANOVA) to explore possible relationships between individual characteristics and questionnaire score.

## Results

In our experiments, MR technology can be smoothly integrated into surgical workflow. The doctors use the MR system to convert conventional imaging formats into 3D holographic models for visualization with HoloLens, as well as to optimize these holographic models for preoperative and intraoperative use. By overlaying the location of the patient’s lesion with the holographic model helps the surgeon to view and analyse the lesion from multiple angles and levels, and to accurately determine the size, location and adjacent structures. Also, this solution is conducive to doctor-patient communication, surgical design optimization, accurate intraoperative positioning and navigation, and the improvement of long-distance surgical consultations. To a certain extent, the visualization effect based on MR technology improves the quality of medical services, reflects humanistic care, and achieves better treatment results. The following are illustrative cases for which the orthopaedics surgery by MR technology was particularly effective.

### Case 1: Simpler, More Accurate, and Smoother Communication

**FIGURE 1:**
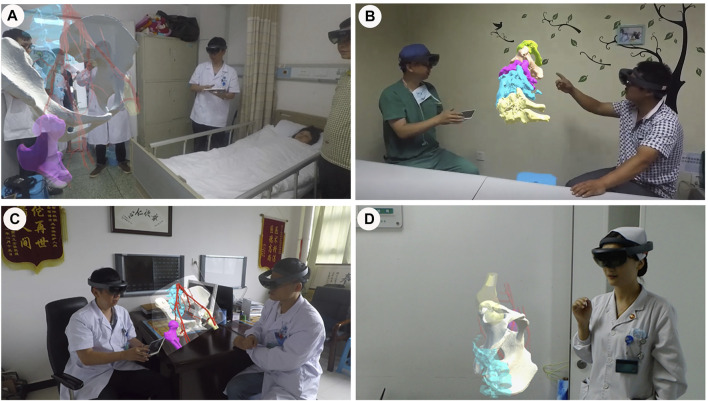
Applications of mixed reality in preoperative communication. **(A,B)**. Effective doctor-patient communication is conducted preoperatively through mixed reality technology. **(C)**. Surgeons discuss technical schemes through mixed reality. **(D)**. Communication between doctors and nurses through mixed reality.

Before starting certain orthopaedic surgeries, we apply the MR system to construct 3D holographic fracture models, where each model could fully and stereoscopically show detailed information of the patient’s fracture. The spatiotemporal awareness inherent in MR overhauls the ill-posed communication between the surgeon, staff, and information. From a patient’s point of view, viewing holograms by HoloLens glasses enables the patient to establish an intuitive understanding of his (or her) fracture before the surgery (Figures 1A,B). This understanding of the medical conditions is useful not only for patients but also for their families. The holograms are also convenient for surgeons to directly and accurately point out the necessity and the potential risks that might be associated with an operation, increase the confidence of the patients and their families in the surgeon’s decisions, and enhance the surgical treatment outcomes. To a certain extent, these holograms alleviate possible tensions in the doctor-patient relationship that might arise due to the medical information asymmetry between doctors and patients. From a doctor’s perspective, viewing 3D lifelike dynamic holograms provides an intuitive communication platform for doctors in the same discipline or in different disciplines (Figures 1C,D). As well, the MR hologram deepens the doctor’s understanding of the fracture degree and type. This thereby provides a powerful tool for formulating a suitable treatment plan.

### Cases 2–3: Intraoperative Guidance

**FIGURE 2:**
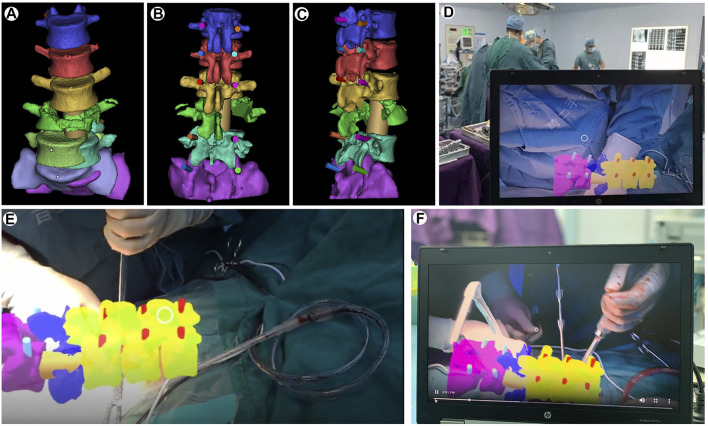
Mixed-reality-guided spinal fracture surgery. **(A)**. Spinal fracture 3D reconstruction with spiral CT. **(B,C)**. Computer simulations for pedicle screw placement. **(D,E,F)**. Mixed-reality-guided osteotomy and pedicle screw implantation.

Case 2. A 29 year-old male patient had a severe fall which resulted in a burst fracture of the lumbar vertebra and a spinal cord injury. Prior to surgery, the fracture types and the internal fixation approaches were analyzed by CT structural data. Then, these DICOM data were reconstructed and segmented by StarCloud workstation. Different colors were used to distinguish different structures to obtain a “3D fracture digital model”, then simulate pedicle screw placement on this model (Figures 2A–C). The ideal trajectories for pedicle screw placement are achieved by eliminating the structural hierarchy, and compared with the measurement data on the workstation. Then the reconstructed 3D model is injected into HoloLens glasses, and project the hologram. During the operation, after the holograms had been aligned and rotated by gesture control or voice, the 3D holographic model is projected on the above of the surgical area to guide the surgeon to design the incision size (Figure 2D). Following separation of the paraspinal muscles from the vertebra, the real position of the injured spine was overlaid on the virtual 3D digital model through the HoloLens glasses to update the surgeon’s perspective. The manual rigid co-registration between the holograms and the surgical vertebrae is accomplished according to the four bony landmarks (the spinous process, lamina, and the superior and inferior articular processes adjacent to the pedicle) selected before the operation (Figures 2E,F). Subsequently, under the guidance of the MR hologram, the pedicle screw is placed from the preoperatively defined ideal pedicle entry point to the end point. The rest of the surgery proceeded in standard fashion that a pre-bent connection rod was attached and the height of the vertebral body was restored after proper longitudinal distraction and reduction, and then tighten the nut. Finally, the bone graft procedure was completed with a titanium mesh cage. All procedures were completed successfully and intraoperative fluoroscopy showed satisfactory reduction.

The patients experienced significant pain relief and neurological improvement immediately after surgery. At the 3 months follow-up, he fully recovered normal neurological function.

**FIGURE 3:**
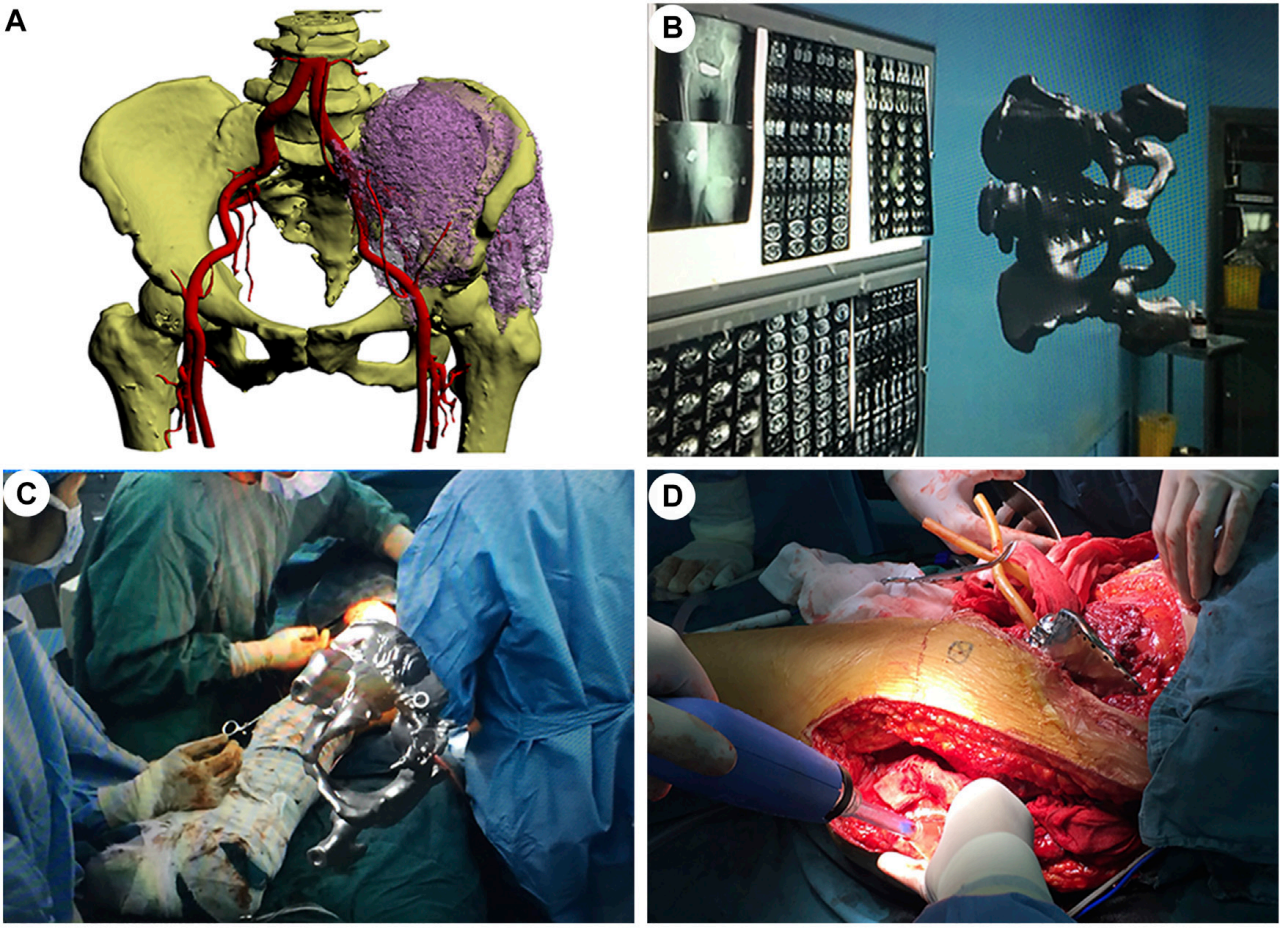
Mixed-reality-based orthopaedic surgery with a personalized 3D-printed prosthesis. **(A)**. A 3D reconstructed model of the patient’s pelvis showing the extent of the tumor and its important surrounding structures. **(B)**. Mixed-reality representation of the pelvic tumor. **(C)**. Mixed-reality-guided tumor removal. **(D)**. Successful placement of the personalized 3D-printed prosthesis.

Case 3. A 61 year-old female patient was diagnosed with osteosarcoma of the left pelvis. Extensive resection and reconstruction were performed. We planned osteotomy assisted by MR hologram and reconstruction using a personalized titanium alloy hemi-pelvic prosthesis. Preoperatively, CT and MRI data are collected and fused to create a 3D virtual model, which is then given different colours and transparency using its PC client. This process allows data to be accessed wirelessly *via* HoloLens glasses (Figures 3A,B). The location, shape and size of the tumour and its surrounding anatomy in the model are consistent with the findings of the patient’s imaging data. Moreover, the personalized hemi-pelvic prosthesis was digitally designed and 3D printed according the morphology of unaffected side hemi-pelvis which subsequently implanted in surgery to reconstruct the pelvis. During surgery, the hologram is projected onto the patient’s body surface using a head-mounted device. The MR overlay injected into HoloLens helped verify regional anatomy including tumor location allowing for optimizing resection and preservation of key structures. After having verified the overlay’s accuracy by using marker points and characteristic markers on the body surface (anterior superior iliac spines and posterior superior iliac spines), an incision was made in the outward aspect of the pelvis to reach the ilium. With increased awareness provided by the overlay, the tumor on the left iliac was fully dissected and exposed (Figures 3C,D). Subsequently, in tumor and pelvic resection phase, the osteotomy is completed according to the preoperative design, guided by the 3D hologram, and the personalized hemi-pelvic prosthesis was implanted and fixed to reconstruct the pelvis. Finally, the incision was washed with physiological saline and then sewn up.

The patient had normal anesthesia recovery and could move the left leg slightly. Postoperative X-ray scan demonstrated the removal of the tumor and a good position of the prosthesis.

### Case 4: Surgical Navigation

**FIGURE 4:**
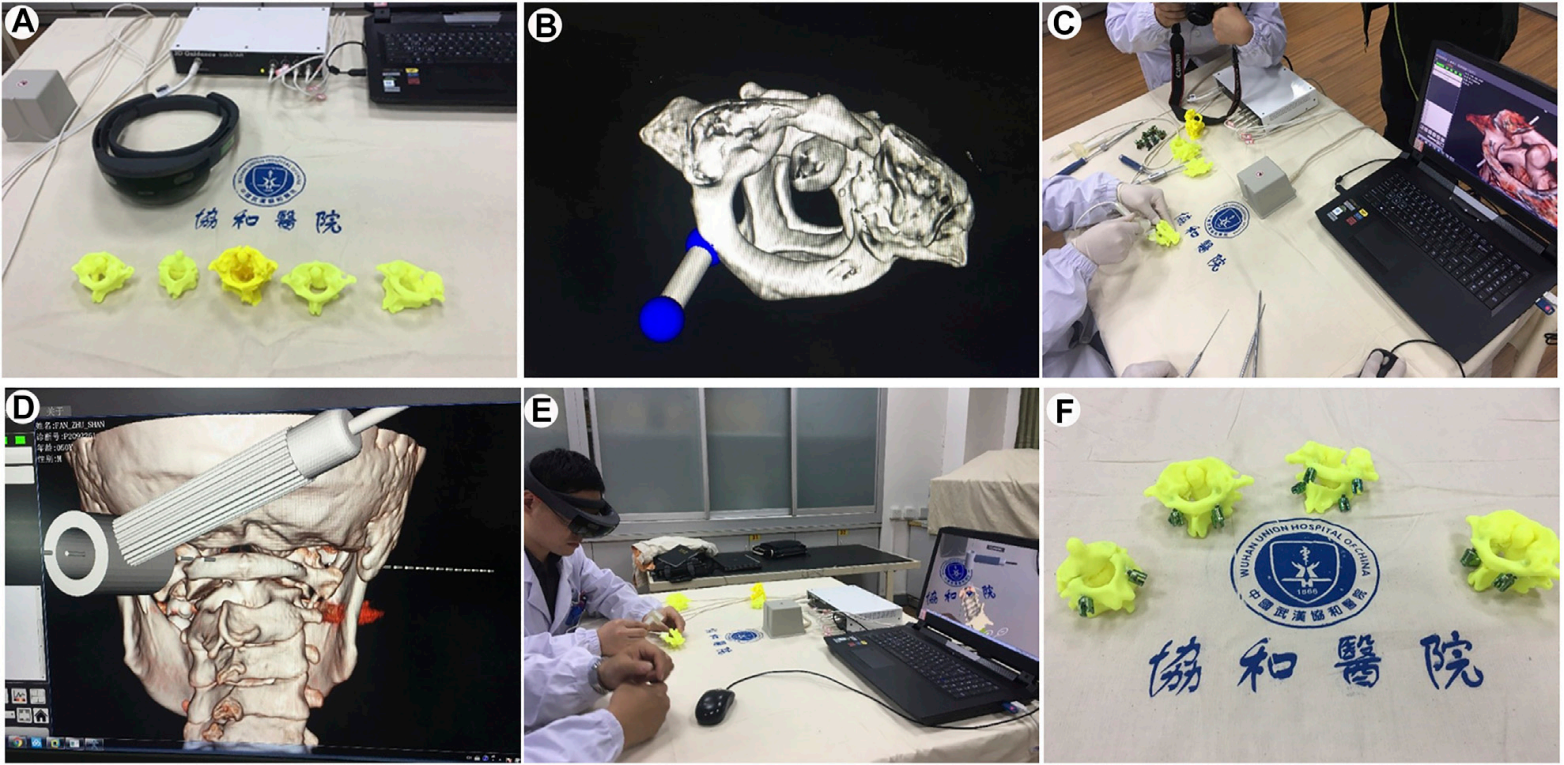
Mixed-reality-based navigation for the atlanto-axial pedicle screw placement. **(A)**. 3D printed models for atlanto-axial fractures and dislocations. **(B)**. Computer simulations of the direction and angle settings for pedicle screw placement. **(C,D,E)**. Pedicle screw placement in the atlanto-axial fracture models under the guidance of the MR-based navigation system. **(F)**. Precisely-inserted pedicle screws into the atlanto-axial fracture models.

In this study, we developed a MR-assisted orthopaedics surgery method using the electromagnetic tracking system. First, raw CT data of C1/2 fracture was obtained from the picture archive and communication system (PACS) of Wuhan Union Hospital. Then, the 3D model of the C1/2 fracture was reconstructed using MIMICS 19.1 software, then producing 3D printing (Figure 4A). The fracture-type was analyzed and a computer simulation of pedicle screw placement was performed, including one or more surgical trajectories and corridors (Figure 4B). Three anatomical bony landmarks were identified on the preoperative model and referenced during surgery. The specific surgical navigation steps were as follows. First, the TrakSTAR tracking tool (sensor) was installed on the 3D printed model as a reference coordinate system. The 3D position of the tracked pointing tools in the physical space of the 3D printed model was tracked by the TrakSTAR tracking tool in order to mark the point of entry and the trajectory of the screw placement and the information was input into the computer (Figures 4C,D). Then, each model with planned internal fixation was imported to HoloLens glasses. During surgery, the virtual model was utilized in conjunction with the electromagnetical tracking system for enhanced guidance via MR hologram overlay with the 3D-printed model. The surgeon observes the virtual models and simulated the operative procedures through HoloLens glasses. The registration between the 3D-printed model and 3D hologram were performed by point-to-point matching of the three preoperatively defined landmarks. To verify overlay accuracy, alignment of the hologram with its physical counterpart was visually confirmed by the surgeon (Figure 4E). The holographic model of the fracture, the tracked entry point and trajectory of pedicle screw on it were shown on the PC screen and HoloLens glasses. Subsequently, the screws were sequentially placed into the printed model under the guidance of MR hologram according to the tracked trajectory and entry point (Figure 4F). The postoperative radiological results showed that the screws were well positioned and consistent with the preoperative planning.

### Cases 5–6: Mixed-Reality-Assisted Telesurgery

**FIGURE 5:**
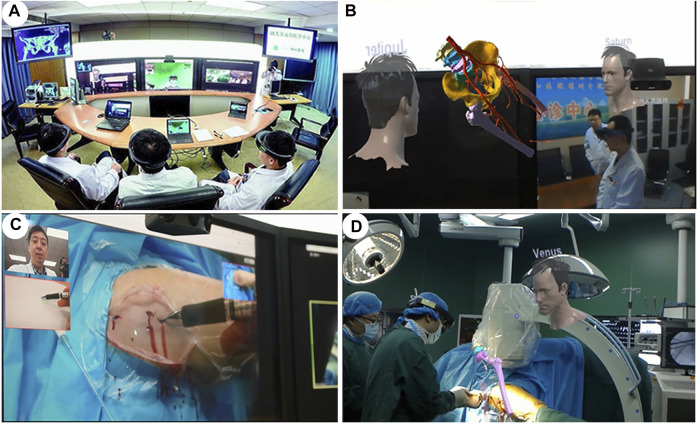
An overview of the cloud-based mixed-reality telesurgery platform. **(A,B)**. Surgeons in different places conduct MR-based preoperative planning. **(C,D)**. Real-time intraoperative guidance of an orthopaedic surgery by remote experts.

Case 5. A 59 year-old female patient was found to have a hip fracture in Xinjiang Bozhou Hospital, a primary hospital (more than 1,300 km away from our main hospital). The patient’s CT data was preoperatively imported into the StarCloud workstation, and a 3D model of the fracture was rapidly reconstructed and stored in the cloud-based MR collaboration platform. Doctors at different locations exploited this model to carry out preoperative discussions, intraoperative guidance and other operations. Preoperatively, with the support of MR and 5G technology, the holographic images of anatomy in the MR glasses of the surgeon are transmitted in real time to the MR glasses of the experts in long distance. By this way, the experts at Wuhan were able to conduct preoperative physical examination, preoperative assessment and preoperative discussions with surgeons and patients through remote communication (Figures 5A,B). Percutaneous screw fixation (minimally invasive surgery) was planned and performed. Intraoperatively, the experts in Wuhan were projected to the operating room in Xinjiang profit from the application of augmented reality technology combined with 5G technology. The remote specialist was able to share what the surgeons in the operating room sees and hears and to communicate with them in real time, as well as direct the surgical incision on a tablet screen with a guide pen, which the surgeon can then see on his own screen (Figure 5C). The MR holographic model is then projected directly onto or above the operating site during multiple stages of the procedure, including positioning, skin incision and screw placement, providing an anatomical reference for the surgeon to be able to quickly and accurately determine the insertion point and angle of the screw. (Figure 5D). A total of three screws were implanted and intraoperative X-ray showed good internal fixation. After surgery, the patients returned to the ward without complains of discomfort. The telesurgeons were also asked to provide a rating for understanding of information communicated during the procedure.

**FIGURE 6:**
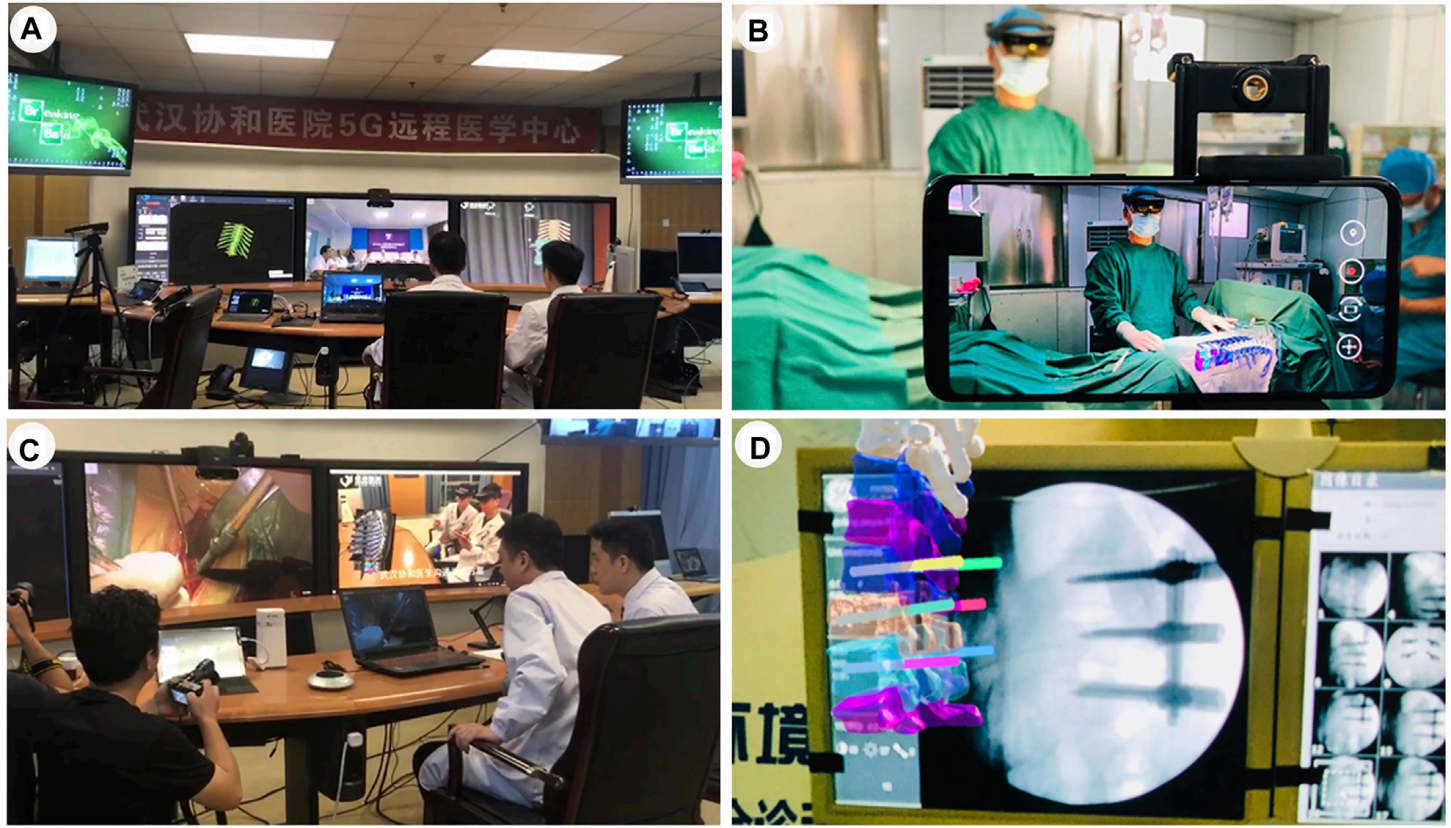
A cloud-based 5G-network mixed-reality platform for telesurgery. **(A)**. The patient’s CT data was imported into Mimics, and a 3D model of the fractured spine was rapidly reconstructed. **(B)**. During the surgical procedure, doctors from different places shared MR holograms in real time through a 5G network. **(C)**. Real-time intraoperative guidance by remote experts. **(D)**. Pedicle screws inserted with high precision into the T12 fracture.

Case 6. A 76 years old female patient was admitted to the Xianfeng County People’s Hospital, a primary care hospital over 600 km from our main hospital. She had suffered a T12 burst fracture in an accident and required immediate surgery. Due to the lack of local medical resources, such an operation was not feasible. Through careful early planning, we used a cloud-based MR platform and 5G network technology to assist the surgeon with remote surgery. 5G communications enabled remote holographic projection images from Wuhan to Xianfeng in Hubei. Specifically, holographic surgical planning, remote surgical guidance, surgical collaboration and remote communication between surgeons were all completed. Pre-operatively, real-time communication between surgeons and remote specialists for pre-operative discussions was carried out through the cloud platform and 5G technology. 3D virtual models were generated and a simulation of screw placement procedures were performed (Figure 6A). During the surgical intervention, the surgeon incorporates the position and orientation of the pre-operative images more effectively into the surgical workflow by moving, zooming or transferring the images close to the surgical site (Figure 6B). At the same time, the surgeon and the specialist communicate in real time *via* the audio-visual system, providing both parties with the same surgical images and thus surgical guidance (Figure 6C). Finally, the surgeries were successfully performed, intraoperative fluoroscopy showed satisfactory reduction (Figure 6D). After surgery, similar questionnaire procedure was performed.

### National Aeronautics and Space Administration Task Load Index and Likert-Scale Questionnaire

**FIGURE 7:**
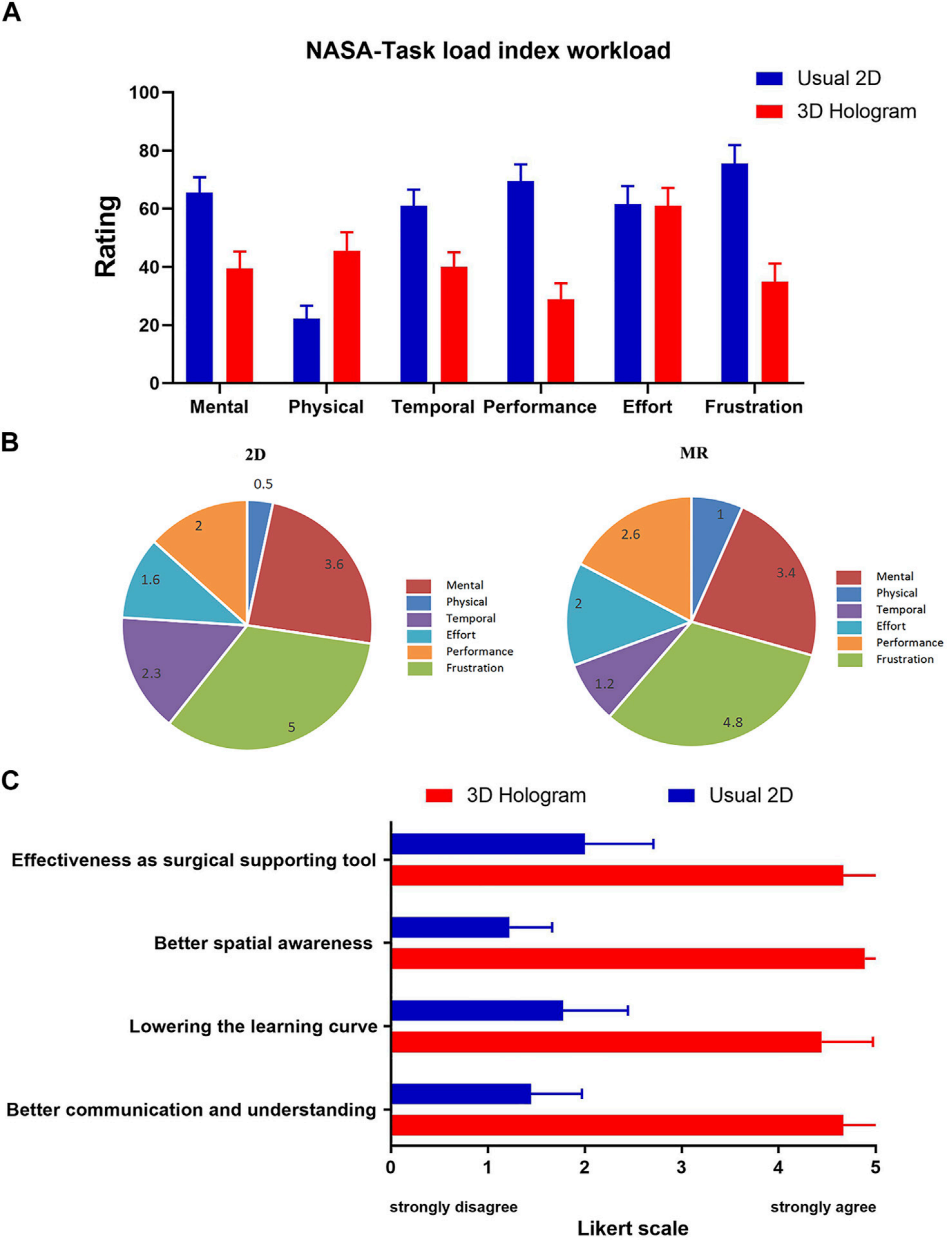
The National Aeronautics and Space Administration-Task Load Index (NASA-TLX) and Likert-scale Questionnaire Scores. **(A)**. MR scored significantly higher “physical demand” than usual 2D support. And, MR scored significantly lower “mental”, “temporal,” “performance” and “frustration.” **(B)**. Weighting of each scale. “Frustration” and “mental” were considered important factors in MR support. **(C)**. Surgeons perceptions of both modes and were rated on a five-point Likert scale from 1 to 5 (1 indicates completely disagree; 2, disagree; 3, neutral; 4, agree; and 5, completely agree).

We evaluated the workload of both conventional 2D image data and MR 3D hologram for surgical support using the NASA Task Load Index (Table 1). As shown in Figure 7, the 3D hologram group received significantly lower “mental,” “temporal,” “performance,”, and “frustration” scores; however, they received significantly a significantly higher “physical demand” rating than usual 2D support. The main reason is that participants need to wear a mixed reality headset, but the weight of the mixed reality glasses was acceptable (Figure 7A). In weighting of each scale, “frustration” and “mental” loads were considered important factors in both technological supports (Figure 7B). The Likert-scale questionnaire revealed that the 3D hologram group had superior results compared to the 2D group (understanding and communicating: 2D group median score 1, IQR 1–2 versus MR hologram group median 5, IQR 4–5; *p* <0.001; spatial awareness: 2D group median score 1, IQR 1-2 versus MR group median 5, IQR 5–5; *p* <0.001; lowering the learning curve: 2D group median score 2, IQR 1–2 versus MR hologram group median 4, IQR 4–5; *p* <0 .001; Effectiveness as surgical supporting tool: 2D group median score 2, IQR 1.5–2.5 versus MR group median 5, IQR 4–5; *p* <0.001; Figure 7C, Table 1).

**TABLE 1:** NASA Task Load Index scores, and Likert scale questionnaire scores.

Category	2D group, mean (SD)	2D group, median (IQR)	3D holographic group, mean (SD)	3D holographic group, median (IQR)	*p* value
NASA Task Load Index scores					
Mental	65.56 (5.27)	65 (60–70)	39.44 (5.83)	40 (35–42.5)	<0.001
Physical	22.22 (4.40)	20 (20–25)	45.56 (6.35)	45 (40–50)	<0.001
Temporal	61.11 (5.64)	60 (60–65)	40.00 (5.00)	40 (37.5–45)	<0.001
Performance	69.44 (5.83)	70 (65–72.5)	28.89 (5.46)	30 (25–30)	<0.001
Effort	61.67 (6.12)	60 (60–67.5)	61.11 (6.01)	60 (57.5–65)	>0.99
Frustration	75.56 (6.35)	75 (70–80)	35 (6.12)	35 (30–40)	<0.001
Likert-scale questionnaire scores					
Better understanding and communication	1.44 (0.53)	1 (1–2)	4.67 (0.5)	5 (4–5)	<0.001
Lowering the learning curve	1.78 (0.67)	2 (1–2)	4.44 (0.53)	4 (4–5)	<0.001
Better spatial awareness	1.22 (0.44)	1 (1–2)	4.89 (0.33)	5 (5–5)	<0.001
Effectiveness as surgical supporting tool	2 (0.71)	2 (1.5–2.5)	4.67 (0.5)	5 (4–5)	<0.001

## Discussion

**FIGURE 8:**
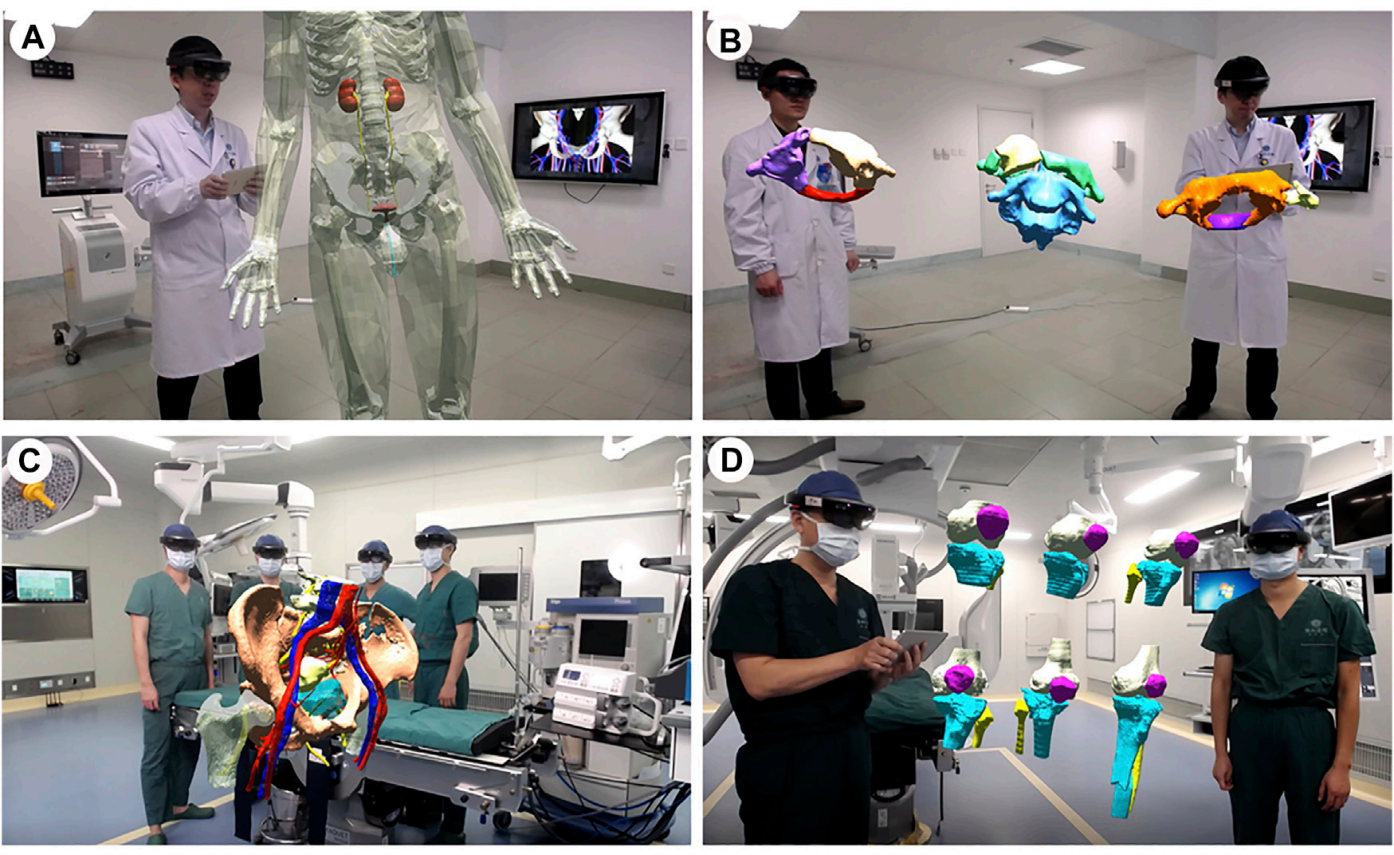
Applications of mixed reality in teaching and training. **(A)**. Building an interactive virtual 3D human body. **(B)**. Classification of the atlantoaxial dislocations. **(C)**. The pelvic fractures and the surrounding structural anatomy. **(D)**. The Schatzker classification of the tibial plateau fractures.

To the best of our knowledge, this study is the first case series detailing the clinical-use workflow of MR hologram in different scenarios during orthopaedics surgery. Similar to previous studies (Bowyer et al., 2010; Craig et al., 2010; Drake et al., 2014; Moro et al., 2017; Ferro et al., 2019; Condino et al., 2021a), we initially used mixed reality technologies for conventional applications of teaching and training, where MR-HMDs are typically put on for intuitive demonstrations and simulations in anatomy courses. However, we have come to realize a great MR potential in analyzing bone fractures, particularly in displaying their 3D structures, and understanding their types and degrees. In our work, we use MR holographic imaging to visualize the 3D fracture structures, render fractures separately, and upgrade fracture observations from the traditional 2D level to the 3D level. While conventional 2D imaging techniques play an essential role in the diagnostic process and preoperative planning of orthopedic surgery, MR hologram could enable 3D, more realistic, and accurate presentation of anatomy. This not only improves the understanding of complex anatomy, but also aids in structural surgical interventions (Kersten-Oertel et al., 2013) (Figure 8). Therefore, having developed a suitable workflow, we investigated the use of MR holograms in the orthopaedics surgical environment. We show that MR technology has a unique advantage in doctor-patient communication, preoperative planning, image-assisted surgery, and remote surgical consultation.

For any surgery, good doctor-patient communication and meticulous preoperative planning is crucial for optimized surgical treatment. Preoperative conversations are not only important as a communication channel with patients and their families, but also represent a necessary measure for preventing medical disputes, and an embodiment of respecting a patient’s right to know (Chuah et al., 2013). Due to the complexity of the anatomy, the high specialization of the surgery, the limitation of their knowledge and cognition levels, patients and their families often do not fully understand their illness and the treatment options. Compared to traditional two-dimensional diagrams (Duff et al., 2010), holographic modality provides the user with a 3D and more realistic view of the surgical target. By sharing and interpreting MR holographic models with 3D realistic dynamic characteristics and using these models to demonstrate surgical procedures, surgeons improve the patient’s subjective understanding of the illness and surgical plan, thus alleviating the information asymmetry between doctors and patients, and making the communication process simple and efficient. (Prochaska et al., 2016). Moreover, careful preoperative planning will contribute to achieve high levels of precision and avoid complications (Kumar et al., 2020). By using HMDs, medical staff can understand complex anatomical structures as well as individual pathologies in a more comprehensible and intuitive manner. (Devoto et al., 2019). For the visualization of anatomical structures in complex surgical interventions, MR applications simulate 3D images and reduce the offset between working space and visualization allowing for improved spatial-visual approximation of patient and image, it is good for surgeons to make the correct operation plan. Furthermore, preliminary studies have also shown that MR offers an improved training modality for orthopedic residents. A similar study by Condino et al. explored a mixed-reality platform using HoloLens, demonstrating its application as a hybrid training system for orthopedic open surgery with a reduced learning curve (Condino et al., 2021a).

In an intraoperative setting, surgeons could access anatomical information of the patient through the MR system and HoloLens glasses, then superimpose virtual holographic elements onto the actual superficial anatomy of the patient while on surgical table, in real time. This holographic guidance enables the surgeons to obtain a high-dimensional “perspective” operation area and a better spatial awareness, which will improve accuracy, safety, and efficiency of the operation. (Kersten-Oertel et al., 2013; Wu et al., 2018b). Use of a hologram is also guaranteed to not contaminate the operating field and avoids the distraction caused by the surgeons constantly having to divert their gaze to a separate screen during the procedure (Condino et al., 2021b), and the unnecessarily prolonged operation time that ensues. Moreover, Saito et al. also reported that during the surgical operations, last-minute simulations can be realized by sharing holograms. This can fully relieve the psychological pressure of the surgeons (Saito et al., 2020). These may account for MR hologram scored significantly lower “mental,”“temporal,” “performance,” and “frustration” workload than traditional 2D support. In addition, we propose an MR navigation system based on electromagnetic signals to orthopaedics surgery that does not require extensive equipment. On the one hand, compared with our previous application of 3D-printed guide technology (Wu et al., 2017), the MR-based navigation can not only be used with stable C1/2 fractures, but it can also be used in cases of severe spinal column fractures. On the other hand, in comparison to the CT-guided navigation, the MR-based navigation reduces the risk of radiation exposure while allowing lateral, anteroposterior (AP), axial, and trajectory views (Kovanda et al., 2015). Nevertheless, the MR-based navigation system is not a substitute for the anatomical knowledge and experience of the surgeons, but it is just an auxiliary assistive tool similar to other image-guided methods.

Beyond conventional surgery uses, MR also offers a new strategy for remote surgical consultation. Traditional telemedicine systems have image processing modules, audio transmission modules, and video frame capture modules. Telemedicine specialists obtain surgical images by capturing frames, marking images on the screen, then sending the images back to the operation scene with both audio and video guidance (Batsis et al., 2019). Indeed, this is a non-real-time guidance system, with a certain time lag. Through the MR-based telesurgery system, this situation is radically changed. The emergence of the holographic mixed-reality (MR) technology is likely to be a key milestone in the development of telehealth. In the considered teleconsultation cases, we used the 5G network for full-HD real-time interaction and live broadcast. The 5G network has the advantages of a high broadband, a low delay, high-speed coding, and edge computing. The combination of the advantages of the 5G network and the MR real-time interaction enabled real-time transmission, visual expression, and accurate understanding of 3D model information. This combination also breaks through the physical presence limitations, especially in line with the telemedicine requirements. With the 5G network, surgical operations can be accompanied by high-throughput calculations, analysis of MR holographic stereo images, and live cloud video over 4K. These system features enable off-site experts and surgeons to share MR holograms and interact in real time, in order to complete operations successfully and with the highest quality. As well, the effective combination of these technologies can realize the sharing of high-quality medical resources, thereby alleviating the imbalance in medical resource availability across various geographic regions.

### Challenge and Future Development

As a promising novel technology, there are some yet unresolved issues for MR. First, the registration accuracy still needs improvements. The manipulation of the surgeon, respiratory motion, and the interaction with the surgical instruments orthopeadics surgery could affect the registration. (Wei et al., 2019). Future directions for MR research in surgery need to be focused on creating systems that are capable of accurate automated image overlay, in order to limit the potential for error. Second, the resolution of the HoloLens glasses is not high enough to render high-quality and high-fidelity 3D images. Also, a horizontal 30-degree field of view prevents users from obtaining a perfect experience. Third, the perceptual issues, intrinsic to standard optical see-through HMDs, due to mismatched accommodation between the virtual content and the real-world scene (Condino et al., 2018; Condino et al., 2020). Fourth, wearing HoloLens glasses for a long time may cause human eye discomfort and even dizziness. Last, human-computer interaction needs to be improved (Hough et al., 2015). So far, most MR-based systems only track the user’s head and hands, while ignoring other perception and interaction channels. This limited sensing technology seriously restricts the design and development of multi-channel interactive MR systems.

Notably, with recent advancements in display and wearable technologies, users will enjoy better experiences with 3D holographic models. Moreover, due to the outbreak of the 2019 novel coronavirus (COVID-19), MR-based telemedicine systems are expected to be more widely used in the next 5 years. As the model registration and interactivity issues are better addressed, the MR technology can be expected to be applied routinely in the perioperative period within 10 years. This can be achieved through the seamless combination with advanced technologies of 5G, artificial intelligence, and cloud computing.

### Limitations

There are several limitations in this study. For instance, it is a case study rather than a randomized controlled trial that explores the potential of mixed reality (MR) technology in the visualization of orthopedic surgery, and the number of cases was small. Randomized studies with a larger number of cases are needed to confirm the results. Moreover, thorough comparative analysis of MR technology utility and accuracy during specific stages of surgery should be performed.

## Conclusion

In this study, our aim was to demonstrate the potential use of MR technology for image-guided orthopeadics surgery and provide information to surgeons for implementing such technology. Although still in its infancy, this technology might herald a revolution in the way we perform surgery. The integration of MR technology in surgery is clearly one of the future development directions in orthopaedics.

## Data Availability

All datasets presented in this study are included in the article/Supplementary Material.

## References

[B1] Al Janabi H. F. Aydin A. Palaneer S. Macchione N. Al-Jabir A. Khan M. S. (2020). Effectiveness of the HoloLens Mixed-Reality Headset in Minimally Invasive Surgery: a Simulation-Based Feasibility Study. Surg. Endosc. 34 (3), 1143–1149. 10.1007/s00464-019-06862-3 31214807 PMC7012955

[B2] Batsis J. A. DiMilia P. R. Seo L. M. Fortuna K. L. Kennedy M. A. Blunt H. B. (2019). Effectiveness of Ambulatory Telemedicine Care in Older Adults: A Systematic Review. J. Am. Geriatr. Soc. 67 (8), 1737–1749. 10.1111/jgs.15959 31066916 PMC6684409

[B3] Bowyer M. W. Hanson J. L. Pimentel E. A. Flanagan A. K. Rawn L. M. Rizzo A. G. (2010). Teaching Breaking Bad News Using Mixed Reality Simulation. J. Surg. Res. 159 (1), 462–467. 10.1016/j.jss.2009.04.032 19665731

[B4] Brigham T. J. (2017). Reality Check: Basics of Augmented, Virtual, and Mixed Reality. Med. Reference Serv. Q. 36 (2), 171–178. 10.1080/02763869.2017.1293987 28453428

[B5] Brun H. Bugge R. A. B. Suther L. K. R. Birkeland S. Kumar R. Pelanis E. (2019). Mixed Reality Holograms for Heart Surgery Planning: First User Experience in Congenital Heart Disease. Eur. Heart J. Cardiovasc. Imaging 20 (8), 883–888. 10.1093/ehjci/jey184 30534951

[B6] Carbone M. Piazza R. Condino S. (2020). Commercially Available Head-Mounted Displays Are Unsuitable for Augmented Reality Surgical Guidance: A Call for Focused Research for Surgical Applications. Surg. Innov. 27 (3), 254–255. 10.1177/1553350620903197 32037972

[B7] Chuah J. H. Lok B. Black E. (2013). Applying Mixed Reality to Simulate Vulnerable Populations for Practicing Clinical Communication Skills. IEEE Trans. Vis. Comput. Graphics 19 (4), 539–546. 10.1109/TVCG.2013.25 23428437

[B8] Chytas D. Nikolaou V. S. (2021). Mixed Reality for Visualization of Orthopedic Surgical Anatomy. World J. Orthop. 12 (10), 727–731. 10.5312/wjo.v12.i10.727 34754828 PMC8554346

[B9] Colomer C. Llorens R. Noé E. Alcañiz M. (2016). Effect of a Mixed Reality-Based Intervention on Arm, Hand, and finger Function on Chronic Stroke. J. Neuroeng. Rehabil. 13 (1), 45. 10.1186/s12984-016-0153-6 27169462 PMC4864937

[B10] Comeau R. M. Sadikot A. F. Fenster A. Peters T. M. (2000). Intraoperative Ultrasound for Guidance and Tissue Shift Correction in Image-Guided Neurosurgery. Med. Phys. 27 (4), 787–800. 10.1118/1.598942 10798702

[B11] Condino S. Carbone M. Piazza R. Ferrari M. Ferrari V. (2020). Perceptual Limits of Optical See-Through Visors for Augmented Reality Guidance of Manual Tasks. IEEE Trans. Biomed. Eng. 67 (2), 411–419. 10.1109/TBME.2019.2914517 31059421

[B12] Condino S. Cutolo F. Cattari N. Colangeli S. Parchi P. D. Piazza R. (2021a). Hybrid Simulation and Planning Platform for Cryosurgery with Microsoft HoloLens. Sensors 21 (13), 4450. 10.3390/s21134450 34209748 PMC8272062

[B13] Condino S. Montemurro N. Cattari N. D’Amato R. Thomale U. Ferrari V. (2021b). Evaluation of a Wearable AR Platform for Guiding Complex Craniotomies in Neurosurgery. Ann. Biomed. Eng. 49 (9), 2590–2605. 10.1007/s10439-021-02834-8 34297263

[B14] Condino S. Turini G. Parchi P. D. Viglialoro R. M. Piolanti N. Gesi M. (2018). How to Build a Patient-specific Hybrid Simulator for Orthopaedic Open Surgery: Benefits and Limits of Mixed-Reality Using the Microsoft HoloLens. J. Healthc. Eng. 2018, 1–12. 10.1155/2018/5435097 PMC6236521 30515284

[B15] Craig S. Tait N. Boers D. McAndrew D. (2010). Review of Anatomy Education in Australian and New Zealand Medical Schools. ANZ J. Surg. 80 (4), 212–216. 10.1111/j.1445-2197.2010.05241.x 20575945

[B16] Devoto L. Muscroft S. Chand M. (2019). Highly Accurate, Patient-specific, 3-Dimensional Mixed-Reality Model Creation for Surgical Training and Decision-Making. JAMA Surg. 154 (10), 968–969. 10.1001/jamasurg.2019.2546 31433467

[B17] Drake R. L. McBride J. M. Pawlina W. (2014). An Update on the Status of Anatomical Sciences Education in United States Medical Schools. Am. Assoc. Anatomists 7 (4), 321–325. 10.1002/ase.1468 24895314

[B18] Duff M. Chen Y. Cheng L. Liu S.-M. Blake P. Wolf S. L. (2013). Adaptive Mixed Reality Rehabilitation Improves Quality of Reaching Movements More Than Traditional Reaching Therapy Following Stroke. Neurorehabil. Neural Repair 27 (4), 306–315. 10.1177/1545968312465195 23213076

[B19] Duff M. Chen Y. Attygalle S. Sundaram H. Rikakis T. (2010). Mixed Reality Rehabilitation for Stroke Survivors Promotes Generalized Motor Improvements. Annu. Int. Conf. IEEE Eng. Med. Biol. Soc. 2010, 5899–5902. 10.1109/IEMBS.2010.5627537 21096934

[B20] Elliott V. de Bruin E. D. Dumoulin C. (2015). Virtual Reality Rehabilitation as a Treatment Approach for Older Women with Mixed Urinary Incontinence: a Feasibility Study. Neurourol. Urodynam. 34 (3), 236–243. 10.1002/nau.22553 24415577

[B21] Ferro A. S. Nicholson K. Koka S. (2019). Innovative Trends in Implant Dentistry Training and Education: A Narrative Review. J. Clin. Med. 8 (10), 1618. 10.3390/jcm8101618 PMC6832343 31590228

[B22] Goo H. W. Park S. J. Yoo S.-J. (2020). Advanced Medical Use of Three-Dimensional Imaging in Congenital Heart Disease: Augmented Reality, Mixed Reality, Virtual Reality, and Three-Dimensional Printing. Korean J. Radiol. 21 (2), 133–145. 10.3348/kjr.2019.0625 31997589 PMC6992436

[B23] Halic T. Kockara S. Bayrak C. Rowe R. (2010). Mixed Reality Simulation of Rasping Procedure in Artificial Cervical Disc Replacement (ACDR) Surgery. BMC Bioinformatics 11 (Suppl. 6), S11. 10.1186/1471-2105-11-S6-S11 PMC3026358 20946594

[B24] Hough G. Williams I. Athwal C. (2015). Fidelity and Plausibility of Bimanual Interaction in Mixed Reality. IEEE Trans. Vis. Comput. Graphics 21 (12), 1377–1389. 10.1109/TVCG.2015.2480060 26394427

[B25] Kersten-Oertel M. Jannin P. Collins D. L. (2013). The State of the Art of Visualization in Mixed Reality Image Guided Surgery. Comput. Med. Imaging Graph. 37 (2), 98–112. 10.1016/j.compmedimag.2013.01.009 23490236

[B26] Kovanda T. J. Ansari S. F. Qaiser R. Fulkerson D. H. (2015). Feasibility of CT-based Intraoperative 3D Stereotactic Image-Guided Navigation in the Upper Cervical Spine of Children 10 Years of Age or Younger: Initial Experience. Ped 16 (5), 590–598. 10.3171/2015.2.PEDS14556 26207668

[B27] Kumar R. P. Pelanis E. Bugge R. Brun H. Palomar R. Aghayan D. L. (2020). Use of Mixed Reality for Surgery Planning: Assessment and Development Workflow. J. Biomed. Inform. 112, 100077. 10.1016/j.yjbinx.2020.100077 34417006

[B28] Lee S. C. Fuerst B. Fotouhi J. Fischer M. Osgood G. Navab N. (2016). Calibration of RGBD Camera and Cone-Beam CT for 3D Intra-operative Mixed Reality Visualization. Int. J. CARS 11 (6), 967–975. 10.1007/s11548-016-1396-1 27059022

[B29] Li G. Dong J. Wang J. Cao D. Zhang X. Cao Z. (2020). The Clinical Application Value of Mixed‐reality‐assisted Surgical Navigation for Laparoscopic Nephrectomy. Cancer Med. 9 (15), 5480–5489. 10.1002/cam4.3189 32543025 PMC7402835

[B30] Li Y. Chen X. Wang N. Zhang W. Li D. Zhang L. (2019). A Wearable Mixed-Reality Holographic Computer for Guiding External Ventricular drain Insertion at the Bedside. J. Neurosurg. 131, 1599–1606. 10.3171/2018.4.JNS18124 30485188

[B31] Lungu A. J. Swinkels W. Claesen L. Tu P. Egger J. Chen X. (2021). A Review on the Applications of Virtual Reality, Augmented Reality and Mixed Reality in Surgical Simulation: an Extension to Different Kinds of Surgery. Expert Rev. Med. Devices 18 (1), 47–62. 10.1080/17434440.2021.1860750 33283563

[B32] Martin G. Koizia L. Kooner A. Cafferkey J. Ross C. Purkayastha S. (2020). Use of the HoloLens2 Mixed Reality Headset for Protecting Health Care Workers during the COVID-19 Pandemic: Prospective, Observational Evaluation. J. Med. Internet Res. 22 (8), e21486. 10.2196/21486 32730222 PMC7431236

[B33] Milgram P. Kishino F. (1994). A Taxonomy of Mixed Reality Visual Displays. IEICE Trans. Inf. Syst. E77-D (12), 1321–1329.

[B34] Moro C. Štromberga Z. Raikos A. Stirling A. (2017). The Effectiveness of Virtual and Augmented Reality in Health Sciences and Medical Anatomy. Am. Assoc. Anatomists 10 (6), 549–559. 10.1002/ase.1696 28419750

[B35] Park B. J. Hunt S. J. Martin C. 3rd Nadolski G. J. Wood B. J. Gade T. P. (2020). Augmented and Mixed Reality: Technologies for Enhancing the Future of IR. J. Vasc. Interv. Radiol. 31 (7), 1074–1082. 10.1016/j.jvir.2019.09.020 32061520 PMC7311237

[B36] Patel T. Ivo J. Faisal S. McDougall A. Carducci J. Pritchard S. (2020). A Prospective Study of Usability and Workload of Electronic Medication Adherence Products by Older Adults, Caregivers, and Health Care Providers. J. Med. Internet Res. 22 (6), e18073. 10.2196/18073 32348292 PMC7298635

[B37] Press V. Meltzer D. Arora V. Prochaska M. (2016). Patient Perceptions of Wearable Face-Mounted Computing Technology and the Effect on the DoctorPatient Relationship. Appl. Clin. Inform. 07 (4), 946–953. 10.4338/ACI-2016-06-LE-0094 PMC5228136 27730249

[B38] Said S. Gozdzik M. Roche T. R. Braun J. Rössler J. Kaserer A. (2020). Validation of the Raw National Aeronautics and Space Administration Task Load Index (NASA-TLX) Questionnaire to Assess Perceived Workload in Patient Monitoring Tasks: Pooled Analysis Study Using Mixed Models. J. Med. Internet Res. 22 (9), e19472. 10.2196/19472 32780712 PMC7506540

[B39] Saito Y. Sugimoto M. Imura S. Morine Y. Ikemoto T. Iwahashi S. (2020). Intraoperative 3D Hologram Support with Mixed Reality Techniques in Liver Surgery. Ann. Surg. 271 (1), e4–e7. 10.1097/SLA.0000000000003552 31425293

[B40] Sauer I. M. Queisner M. Tang P. Moosburner S. Hoepfner O. Horner R. (2017). Mixed Reality in Visceral Surgery. Ann. Surg. 266 (5), 706–712. 10.1097/SLA.0000000000002448 28767561

[B41] Schuster-Amft C. Eng K. Lehmann I. Schmid L. Kobashi N. Thaler I. (2014). Using Mixed Methods to Evaluate Efficacy and User Expectations of a Virtual Reality-Based Training System for Upper-Limb Recovery in Patients after Stroke: a Study Protocol for a Randomised Controlled Trial. Trials 15, 350. 10.1186/1745-6215-15-350 25194928 PMC4167274

[B42] Tepper O. M. Rudy H. L. Lefkowitz A. Weimer K. A. Marks S. M. Stern C. S. (2017). Mixed Reality with HoloLens. Plast. Reconstr. Surg. 140 (5), 1066–1070. 10.1097/PRS.0000000000003802 29068946

[B43] Wei P. Yao Q. Xu Y. Zhang H. Gu Y. Wang L. (2019). Percutaneous Kyphoplasty Assisted With/without Mixed Reality Technology in Treatment of OVCF with IVC: a Prospective Study. J. Orthop. Surg. Res. 14 (1), 255. 10.1186/s13018-019-1303-x 31395071 PMC6686364

[B44] Wu X. Liu R. Yu J. Lu L. Yang C. Shao Z. (2017). Deviation Analysis for C1/2 Pedicle Screw Placement Using a Three-Dimensional Printed Drilling Guide. Proc. Inst. Mech. Eng. H 231 (6), 547–554. 10.1177/0954411916680382 28056709

[B45] Wu X. Liu R. Yu J. Xu S. Yang C. Shao Z. (2018a). Mixed Reality Technology-Assisted Orthopedics Surgery Navigation. Surg. Innov. 25 (3), 304–305. 10.1177/1553350618771413 29701134

[B46] Wu X. Liu R. Yu J. Xu S. Yang C. Yang S. (2018b). Mixed Reality Technology Launches in Orthopedic Surgery for Comprehensive Preoperative Management of Complicated Cervical Fractures. Surg. Innov. 25 (4), 421–422. 10.1177/1553350618761758 30012077

