# Supplementary figures and images for: Applications of Mixed Reality Technology in Orthopedics Surgery: A Pilot Study
Source: Front Bioeng Biotechnol. 2022 Feb 22;10:740507. doi: 10.3389/fbioe.2022.740507 (PMC8902164; doi:10.3389/fbioe.2022.740507)

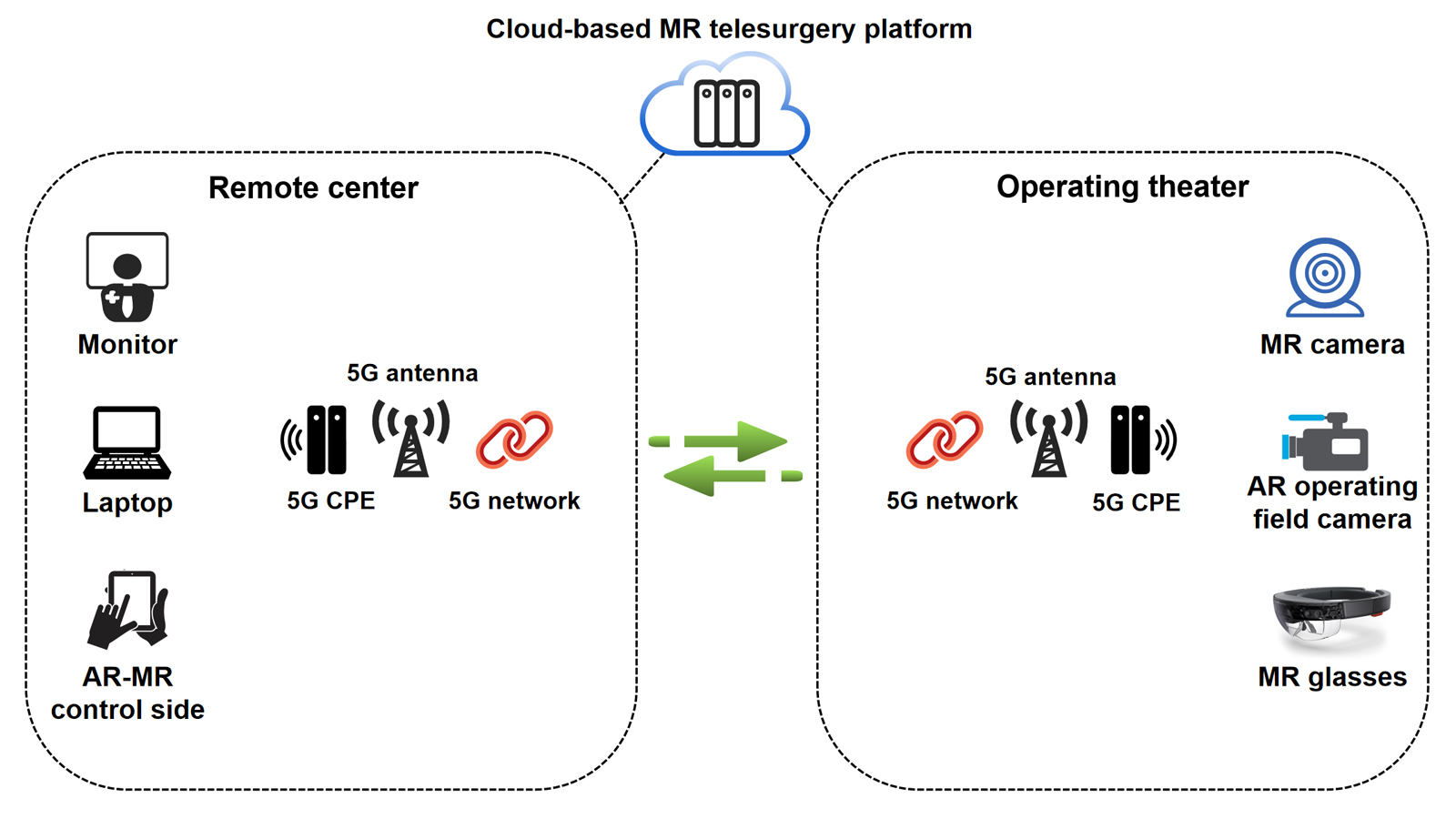

Supplement: Supplementary file 1 [file Image1.JPEG]
